# Target-D: a stratified individually randomized controlled trial of the *diamond* clinical prediction tool to triage and target treatment for depressive symptoms in general practice: study protocol for a randomized controlled trial

**DOI:** 10.1186/s13063-017-2089-y

**Published:** 2017-07-20

**Authors:** Jane Gunn, Caroline Wachtler, Susan Fletcher, Sandra Davidson, Cathrine Mihalopoulos, Victoria Palmer, Kelsey Hegarty, Amy Coe, Elizabeth Murray, Christopher Dowrick, Gavin Andrews, Patty Chondros

**Affiliations:** 1 Department of General Practice, University of Melbourne, Melbourne, VIC Australia; 2 Department of Neurobiology, Care Sciences and Society, Karolinska Institutet, Solna, Sweden; 3 School of Health and Social Development, Deakin University, Burwood, VIC Australia; 4 eHealth Unit, Department of Primary Care and Population Health, University College London, London, UK; 5 Institute of Psychology Health and Society, University of Liverpool, Liverpool, UK; 6 School of Psychiatry, University of New South Wales, Sydney, NSW Australia

**Keywords:** Depression, Clinical prediction tool, Prognosis, Stepped care, General practice, Randomized controlled trial

## Abstract

**Background:**

Depression is a highly prevalent and costly disorder. Effective treatments are available but are not always delivered to the right person at the right time, with both under- and over-treatment a problem. Up to half the patients presenting to general practice report symptoms of depression, but general practitioners have no systematic way of efficiently identifying level of need and allocating treatment accordingly. Therefore, our team developed a new clinical prediction tool (CPT) to assist with this task. The CPT predicts depressive symptom severity in three months’ time and based on these scores classifies individuals into three groups (minimal/mild, moderate, severe), then provides a matched treatment recommendation. This study aims to test whether using the CPT reduces depressive symptoms at three months compared with usual care.

**Methods:**

The Target-D study is an individually randomized controlled trial. Participants will be 1320 general practice patients with depressive symptoms who will be approached in the practice waiting room by a research assistant and invited to complete eligibility screening on an iPad. Eligible patients will provide informed consent and complete the CPT on a purpose-built website. A computer-generated allocation sequence stratified by practice and depressive symptom severity group, will randomly assign participants to intervention (treatment recommendation matched to predicted depressive symptom severity group) or comparison (usual care plus Target-D attention control) arms. Follow-up assessments will be completed online at three and 12 months. The primary outcome is depressive symptom severity at three months. Secondary outcomes include anxiety, mental health self-efficacy, quality of life, and cost-effectiveness. Intention-to-treat analyses will test for differences in outcome means between study arms overall and by depressive symptom severity group.

**Discussion:**

To our knowledge, this is the first depressive symptom stratification tool designed for primary care which takes a prognosis-based approach to provide a tailored treatment recommendation. If shown to be effective, this tool could be used to assist general practitioners to implement stepped mental-healthcare models and contribute to a more efficient and effective mental health system.

**Trial registration:**

Australian New Zealand Clinical Trials Registry (ANZCTR 12616000537459). Retrospectively registered on 27 April 2016. See Additional file 1 for trial registration data.

**Electronic supplementary material:**

The online version of this article (doi:10.1186/s13063-017-2089-y) contains supplementary material, which is available to authorized users.

## Background

### Background and rationale

Depression affects at least 350 million people worldwide [1] and is a leading cause of non-fatal burden of disease [2]. It is costly to individuals in terms of relationships and functioning and to society in terms of direct medical costs and costs due to loss of individual productivity [3]. Despite significant investments in mental health globally, there is no evidence of a reduction in the burden of disease associated with depression [4]. One of the biggest challenges facing mental healthcare systems is the need to develop efficient methods of allocating clinically effective treatment in a cost-effective way to the people that need them most [5].

The majority of depression cases are identified, treated, and followed up in primary care [6]. However, general practitioners (GPs) have been criticized for both under- and over-diagnosing, and treating, depression [7–10]. For example, only 16% of Australians with case level depression or anxiety receive an adequate “dose” of an evidence-based intervention consistent with treatment guidelines [9]. On the other hand, antidepressant prescriptions far outnumber patients for whom such medication is indicated [11].

Multi-country studies report that 24–55% of patients in primary care waiting rooms meet screening criteria for being “probably depressed” [12]. However, among this population of “probably depressed,” a range of illness trajectories exist which contribute to the difficulty experienced by practitioners in making a diagnosis and treatment recommendation [13–18]. It may be that the heterogeneity of clinical presentation which characterizes depression in the primary care setting is leading to the simultaneous problems of both over- and under-diagnosis and treatment.

Currently, there is a mismatch in primary care between patient need and the depression care received, possibly as a result of poor treatment allocation. For example, delivery of intensive interventions to people with minimal or mild symptoms is unnecessarily costly and risks medicalizing normal fluctuations in mood [19]. Conversely, without a targeted intensive intervention, people likely to experience severe and chronic symptoms are likely to experience significant disability, which could have been avoided [20, 21].

Stepped care models, in which patients are, in the first instance, provided with the least time- and resource-intensive intervention that will be effective [22], have been promoted as a potential solution to the problem of poor treatment allocation. Although limited empirical evidence exists as to their effectiveness [23], these models make intuitive sense and feature in both clinical guidelines and policy directives in Australia [24] and the UK [22]. Currently, a key obstacle to the implementation of stepped care models is the lack of effective treatment allocation tools to guide GPs in matching the intensity of treatment to a patient’s needs. In particular, current recommendations focus on matching treatment to the patient’s current symptom severity, rather than patient’s likely course of illness in the future. This is out of step with the management of other conditions (e.g. cardiovascular disease or cancer), which routinely take prognostic factors into account when deciding upon treatment recommendations. Further, it contrasts with calls for research, policy, and practice to make prognosis-based intervention a priority [25]. To date, there has been no quick and systematic way for GPs to identify depression outcomes that a particular person is likely to experience in the future and recommend treatment accordingly.

One option for systematizing treatment recommendations is to use a clinical prediction tool (CPT). CPTs are based on a prognostic model that uses clinical and non-clinical information to estimate an individual’s risk of a specific outcome [26]. The prognostic model is applied in clinical practice using the CPT which stratifies patients into different treatments according to their estimated risk [27]. While CPTs are common in many fields of medicine, they are not readily available for use in mental-healthcare settings. [28]

To address this gap, we wanted to develop a simple, easy-to-use CPT to assist primary care clinicians to triage patients presenting with depressive symptoms and allocate to appropriate treatment. First, we investigated whether an existing prognostic model for depression could be used to build the CPT. We identified several prognostic models that have been developed to predict current [29, 30] or future major depression [31–34] or treatment response [35–37]. However, none of these prognostic models were found to be suitable for incorporating into a CPT which could be easily administered in routine care [38].

Therefore, we developed a novel prognostic model using data from the *diamond* cohort study [39] to predict depressive symptom severity at three months [38]. It comprises 17 items assessing depressive symptom severity at baseline as measured by the Patient Health Questionnaire-9 (PHQ-9) [40]: sex; current anxiety; history of depression; presence of chronic illness affecting daily functioning; self-rated health; living alone; and perceived ability to manage on available income. Based on an individual’s score, he or she is stratified into one of three groups based on predicted depressive symptom scores at three months; namely, minimal/mild (those predicted to have a PHQ score of ≤ 10 at three months), moderate (PHQ > 10 and < 13), and severe (≥13). Cutoffs for the three groups were established during the development of the *diamond* CPT and are explained in full elsewhere [38]. In the intervention being tested in the current study, individuals are then:

1. Presented with feedback reflecting their responses to the CPT;
2. Provided an opportunity to set priorities and reflect on motivation to change; and
3. Presented with an evidence-based treatment recommendation matched to group classification.

The presentation of feedback and treatment recommendation was informed by the principles of motivational interviewing [41] and an iterative development process employing user-centered design principles to ensure the information is presented in a way that is meaningful and engaging for participants [42].

### Objectives

The Target-D randomized controlled trial (RCT) aims to test whether using the *diamond* CPT to tailor treatment recommendations to an individual’s predicted depressive symptom severity is a clinically effective and economically efficient way of reducing depressive symptoms, relative to usual care. This paper presents the study protocol for the Target-D RCT, adhering to the SPIRIT guidelines for intervention trial designs ([43]; see Additional file 2 for SPIRIT checklist).

The primary objective of the Target-D trial is to determine if using the *diamond* CPT to triage individuals with depressive symptoms into symptom severity-appropriate treatment reduces depressive symptoms at three months compared with usual care.

Secondary objectives are to: (1) test whether individuals in the intervention and comparison arms differ in depressive symptom severity at 12 months, quality of life, anxiety symptoms, self-efficacy, and health service use at three and 12 months; (2) determine whether the outcomes differ between the two study arms within each of the three depressive symptom severity groups; and (3) evaluate the cost-effectiveness of the new model of care compared to usual care.

### Trial design

Target-D is a stratified individually RCT with two parallel arms, modelled on the trial undertaken by Hill et al. who tested the stratified management of low back pain [44]. Participants will be randomized to the intervention or usual care arm with 1:1 allocation, stratified by general practice and predicted depressive symptom severity group. Participants in the intervention arm will be categorized into one of three treatment groups according to their *diamond* CPT results; participants in the usual care arm will complete the *diamond* CPT but will not receive feedback, an opportunity for reflection, or a treatment recommendation specific to their predicted depressive symptom severity. An intention-to-treat (ITT) approach will be used in the analysis (explained further below).

## Methods

### Participants, interventions, and outcomes

#### Study setting

The study will be conducted in at least ten general practices in Victoria, Australia (see Additional file 3 for the location of study sites).

### Eligibility criteria

General practices will be eligible if they: see more than 50 adults aged 18–65 years per day; agree to waiting room screening; have a private space available to be used for the Target-D intervention; and have the majority of their GPs willing collaborate with the Target-D team.

Patients attending participating general practices will be assessed for eligibility using a self-report survey delivered via an iPad. Patients will be eligible if they score 2 or more on the two-item version of the Patient Health Questionnaire (PHQ-2) [45] (indicating depressive symptoms but not necessarily a diagnosis of major depressive disorder), are aged 18–65 years, have access to the Internet for the duration of follow-up, have sufficient written English to follow an Internet-based cognitive behavioral therapy (iCBT) program, have not changed depression medication in the past month (if they take such medication), and agree to randomization to either the usual care or intervention arm. They will be ineligible if they are currently taking antipsychotic medication, are regularly seeing or planning to see a psychologist in the next three months, or are currently using an iCBT program. Eligible patients will be asked to provide informed consent online (see Additional file 4) and complete baseline measures prior to completing the *diamond* CPT.

### Intervention

Participants will complete the *diamond* CPT online on a purpose-built study website (henceforth referred to as the Target-D website). They will then be contacted by phone by a trained research assistant (RA) to provide encouragement and support and answer questions as necessary. This phone call will occur within one week of *diamond* CPT completion.

#### Intervention arm

As described above, the intervention being tested comprises feedback on CPT responses, an opportunity to set priorities, and a treatment recommendation (based on predicted depressive symptom severity). Immediately after completing the *diamond* CPT, participants in the intervention arm will see these components displayed on sequential pages of the Target-D website.

The follow-up phone call from an RA will involve a discussion about the treatment recommendation they received, using the results of the *diamond* CPT to tailor the discussion to the individual’s classification. To encourage treatment engagement, this discussion will use motivational interviewing, an approach to conversations that promotes collaboration and aims to strengthen the person’s motivation and commitment to making a change [41].

The recommended treatment for each of the three groups were selected based on a stepped care approach [22], with treatment intensity lowest in the minimal/mild group and highest in the severe group. To select the specific treatments offered to each level of intensity, we examined existing primary care data from the *diamond* cohort study to describe the characteristics, treatment, and service use of individuals stratified to each group. We also reviewed systematic reviews of the evidence relevant to each group and presented these findings to our investigator team to inform treatments offered. Another comprehensive description of the interventions delivered, using the TIDier checklist, will be included in the primary outcome paper as per Hoffmann et al. [46] and CONSORT guidelines.

##### Minimal/mild depressive symptoms at three months

Participants who are likely to have minimal or mild depressive symptoms at three months will be offered self-help and automated follow-up using the myCompass iCBT program which has been shown in randomized trials to be effective in improving outcomes for patients with mild depression [47, 48]. myCompass is an interactive, self-help Internet resource consisting of information, accounts of others’ experiences, treatment modules with home tasks, and mood tracking functions. myCompass uses an internal algorithm to recommend components tailored to participant symptoms and needs. Participants can choose to follow the recommendation or not and may undertake components of the program in any order.

Participants in the minimal/mild group will receive two automated emails from the research team to encourage uptake and adherence to the treatment recommendation. These emails will be sent immediately after the participant receives the recommendation to use myCompass and one week later and are in addition to any correspondence the participant receives from the myCompass program.^1^ Emails will provide participants with the link to myCompass, encouragement to get started, and reminders of some of the benefits of the program. This will mimic what would be feasible in the routine clinical setting.

##### Moderate depressive symptoms at three months

The moderate group will be offered clinician-guided iCBT via the *Worry and Sadness* course in the This Way Up program, which has randomized trial evidence of effectiveness in reducing moderate symptoms of depression [49]. This Way Up comprises six structured online lessons using CBT principles and includes lessons in the form of an illustrated story about someone with depression, printable summaries, and homework assignments, and symptom monitoring at the beginning of each session [49]. Lessons are completed in a linear order and each becomes available five days after the previous lesson is completed.

Target-D will follow standard This Way Up protocol, with participants provided with weekly individualized support via phone/email, until they have completed Lesson Two [49]. Support will include positive encouragement to commence or continue treatment, reiterate the importance of homework completion, and respond to general questions by referring back to program materials. This role will be filled by RAs, in line with evidence supporting the effectiveness of non-clinician provided support to This Way Up users [50]. In keeping with published protocol [49], after the completion of Lesson Two phone contact will be made in response to patient request or a deterioration in condition (defined as an increase of ≥ 5 on the PHQ-9 [51]).

##### Severe depressive symptoms at three months

The severe group will be offered collaborative care, an enhanced form of patient care shown to be effective for treatment of moderate to severe depression in primary care [21, 52–54]. Collaborative care is defined as including four key ingredients: a multi-professional approach to patient care; a structured management plan; scheduled follow-ups; and coordinated communication between health professionals involved in management [21, 55]. Target-D participants involved in collaborative care will receive eight appointments with a trained case manager (CM). The CM role will be filled by a non-mental-health specialist such as a registered nurse. This decision was made as it is in keeping with the role filled by practice nurses in managing other chronic conditions such as diabetes and thus should enhance scalability of the Target-D model of care should it be effective.

The Target-D approach to collaborative care is underpinned by the principles of motivational interviewing, to enhance patient engagement and action. The Target-D CM will receive training in the intervention approach by a qualified psychologist and will receive regular supervision and support from the psychologist and project manager (SF) throughout the trial.

Patients in this group will be reminded of upcoming appointments with the Target-D CM via SMS. After each appointment, patients will receive an email from their CM summarizing their discussion and outlining the actions the patient intends to take to manage his/her mental health. With the patient’s consent, the CM will also send a copy of this email to the treating GP and other professionals involved in the patient’s mental healthcare.

#### Comparison arm

Participants randomized to the comparison arm will access health services as usual. The choice of “usual care” as a comparator was made as the study aims to determine the extent to which the intervention improves (or worsens) patient outcomes relative to standard practice [56]. Participants in this arm will also receive some non-therapeutic attention from Target-D to control for any effect of contact with the study team following completion of the *diamond* CPT; thus, this study arm is referred to as “Usual care plus Target-D” or UC+. UC+ participants will be blinded to their depressive symptom severity group allocation and will not receive a tailored treatment recommendation. Instead, they will see a screen on the Target-D website advising them that they will be asked to provide feedback on: (1) their opinions on research in primary care; and (2) how they normally manage their emotional health and wellbeing. Similar to the procedure for intervention participants, those in the usual care arm will be contacted by phone by an RA within one week of *diamond* CPT completion. The RA will reiterate the importance of the participant’s involvement in the study, ask a series of structured questions about the participant’s views on research and the involvement of their general practice in research, and advise the participant that he or she will be contacted via email in 12 weeks.

#### Modifications

The nature of the study interventions is such that no substantive modifications are anticipated. Patients in the minimal/mild and moderate groups may discontinue using the online program at any time and treatment for those in the severe group may be discontinued at patient request.

If any participant indicates high levels of suicidal ideation during contact with a member of the study team (as indicated by a response of “nearly every day” to question 9 on the PHQ-9: “thoughts that you would be better off dead or of hurting yourself in some way”), regardless of study arm allocation, a standardized suicidal ideation assessment used previously by the study team will be administered and the patient’s GP alerted. This will be reported as an adverse event but is unlikely to result in treatment discontinuation or modification. The assessment will determine if the adverse event was related directly to the intervention or other circumstances not intervention related.

#### Concomitant care

In both the usual care and intervention arms, participants will be permitted to continue any treatment they were engaged with at entry to the trial. Concomitant care will be assessed via self-report questionnaire and routinely collected Government data (see below).

#### Treatment adherence

In the intervention arm, adherence to treatment in the minimal/mild and moderate depressive severity groups will be assessed using website analytics within myCompass and This Way Up (i.e. tracking individual log-ins, access of components, completion of modules and lessons). In the severe group, adherence to the treatment plan will be assessed by the Target-D CM as part of the planned follow-up schedule.

In the control arm, in order to compare “usual care” before and during participation in the trial, information about health service use will be collected at each study assessment (see below).

### Outcomes

Outcome measures will be collected at baseline and three and 12 months post randomization. These time points were selected to balance the benefits of multiple assessments against the risk of unduly burdening participants. They allow us to examine both the immediate and longer-term effect of the intervention and, because they are commonly used in trials of mental-health interventions, will permit comparisons to be drawn with other relevant studies.

#### Primary outcome

The primary outcome is the difference between the two treatment arms in mean depressive symptom severity at three months, controlling for baseline depressive symptom severity. Depressive symptom severity was selected as the outcome measure rather than a clinical diagnosis of major depressive disorder as it is more relevant to the design and delivery of stepped mental healthcare.

#### Secondary outcomes

Secondary outcomes include difference between study arms in mean depressive symptom severity at 12 months and mean mental-health self-efficacy and anxiety at three and 12 months. The cost-effectiveness of the intervention over the study period will comprise an additional secondary outcome.

### Sample size

Sample size calculation was based upon our trial experience, a systematic review of depression trials [55] and current data from the *diamond* study [39]. The primary objective is to test for a standardized effect size of 0.2SD in mean depressive symptoms at three months between the intervention and comparison arms. However, we based our calculations on our planned subgroup analyses because the sample size required would need to be larger to test for difference between study arms within each of the three depressive symptom severity groups than combined. Therefore, we based sample size on detecting a standardized mean difference of 0.2 between arms for the minimal/mild group (given the potential floor effect we anticipate a smaller intervention effect). We hypothesized a standardized effect size of 0.5 in the moderate and severe depressive symptom severity groups as they have room for greater improvement and will receive more intensive treatments.

Based on the CPT development work, we anticipated that 70% of participants will be classified as being likely to have minimal/mild depressive symptoms, 15% as moderate, and 15% as severe depressive symptoms at three months. We used these estimates to extrapolate the total sample size needed to ensure that we had sufficient power for the sub-group analyses. Based on these assumptions, we required 158 (78 in each arm) participants in each of the moderate and severe groups to detect a standardized effect size of 0.5 and 740 (370 in each arm) in the mild/minimal group to detect a smaller standardized effect size of 0.2, with 80% power and 5% significance level for a two-sided test.

This leads to an anticipated sample size of 1056 participants (528 in each arm), which is also sufficient for the primary objective to detect a standardized effect size of 0.2 in the mean PHQ-9 between study arms, with 90% power and 5% significance level. A standardized effect size of 0.2 is equivalent to a mean change of 1.35 points in the mean depressive symptoms assuming a standard deviation on 6.75 (based on *diamond* data). This effect size is in keeping with those found in systematic reviews of interventions to decrease depressive symptom severity in primary care [57].

**Fig. 1:**
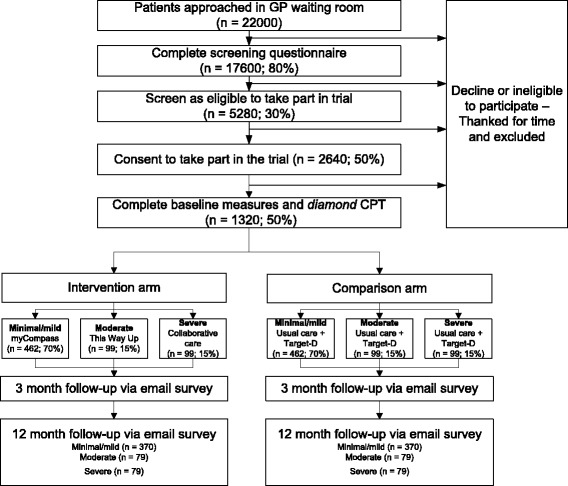
Expected progression of participants through the study

We inflated the required sample size to 1320 to allow for 20% attrition at 12 months. Based upon documented response rates and depressive symptom prevalence gathered from our experience of recruiting participants with depressive symptoms in the primary care setting [39], achieving this sample size at baseline requires that we invite 22,000 adults to complete the screening tool (Fig. 1).

### Recruitment

#### Study sites

We will follow principles of good recruitment by engaging with all stakeholders, branding the Target-D trial, and using a well-developed engagement strategy [58]. We will recruit general practices via our Victorian Primary Care Practice-Based Research Network (VicReN), which has around 200 GP members located in Victoria, Australia. Practices will be contacted by phone and/or email to introduce the study and establish interest. One of the Target-D researchers will then visit interested practices to determine eligibility, provide detailed information about the study, and gain consent to participate. This process will continue until sufficient practices are recruited to obtain the required sample size.

To enhance GP and practice staff engagement in the trial and the activities necessary to make it function, we will be guided by the principles of Normalization Process Theory (NPT) [59]; namely, coherence (meaning of the trial to GPs and staff), cognitive participation (commitment and engagement), collective action (the work GPs and staff do to make the trial function), and reflexive monitoring (GP and staff appraisal of the trial). In each participating practice, GPs and staff will be given a training session clarifying the goals and activities of the trial, in order to instill a sense that the trial is a good idea and worth committing to. In addition, we will clearly outline the trial procedures and how they are likely to affect the work of the practice, with emphasis on how the trial fits with the overall goals of the general practice.

#### Minimizing contamination

Randomizing individuals that were recruited from the same general practice, where the clinician is not blinded to the participant’s study arm or the intervention is implemented at the practice level, may have a greater risk of contamination between intervention and comparison arms than randomizing a group of individuals that do not belong to the same practice [60]. However, the risk of contamination in this trial is expected to be minimal because of several factors. First, recruitment of participants will be conducted in the waiting room by an RA who is not involved in delivering the study intervention and has no access to the allocation schedule. While patients in the waiting room or from the same family or friendship groups may share information, it is unlikely that this will impact the intervention effect as the intervention can only be accessed by permission of the study team. Second, GPs will only be informed of participants allocated to collaborative care treatment. Even if other patients inform their GP that they are participating in Target-D, GPs will not be informed of their treatment allocation nor be able to access study interventions for UC+ patients. Third, the intervention for minimal/mild groups will be via Internet-based programs delivered outside the practice, reducing the potential for practice-based contamination. We will assess the number of UC+ participants registering for these programs to measure the degree of potential contamination. Fourth, the risk that GPs may implement some of the intervention to patients predicted to have severe symptoms and allocated to the UC+ group is small. We anticipate that fewer than ten such participants will be recruited per practice and this small number of patients will be seen by different GPs. We have successfully used a similar approach in a previous RCT in general practice; data from this trial showed very low levels of interaction between comparison participants and the GP during the study time-frame [61].

#### Primary care patients

Potential participants will be alerted to the study via posters and information pamphlets displayed in practice waiting rooms; an awareness raising strategy used by the research team previously. All study materials and procedures were developed and tested with focus groups and individual feedback to ensure they are engaging and user-friendly. Upon completion of the screening survey, eligible participants will enter their name, telephone number, and email address into an online form. They will then be presented with an electronic copy of the plain language statement and provide online consent to participate.

To achieve our required sample size, trained RAs will approach an average of 50 adults per practice per day, taking approximately 440 working days to approach our required 22,000 adults. These numbers are typical of RCTs in primary care and we have achieved similar numbers in previous successful studies [39, 62, 63]. While this number may seem ambitious, several factors contribute to it being achievable. First, we have designed the trial so that participants are recruited by RAs rather than expecting already time-pressured GPs and practice staff to take responsibility for recruitment. Second, the 22,000 patients RAs will approach include all adult patients in the waiting room; RAs or practice staff are not required to identify only those patients who are presenting to the GP for mental health reasons. Finally, we have tried this approach out in a clinic and shown that on weekdays alone an RA can approach at least 50 patients per day and invite 1100 patients per month to the study. Our pilot work has shown that an RA spends only 1 or 2 min with each patient and can comfortably approach 150 patients in a working day. Based on this experience and after accounting for weekend recruitment in some practices, we anticipate participant recruitment to take place over approximately 18 months. Recruitment of participants will continue until the numbers within each depressive symptom severity group have been met.

### Assignment of interventions

#### Allocation

Consent and baseline measures will be collected prior to randomization to minimize reporting and selection bias. When the individual has completed the *diamond* CPT, he or she will be randomly assigned in a 1:1 ratio to the intervention or comparison arm. Randomization will be stratified by general practice and depressive symptom severity group. The allocation sequence will be computer-generated sequentially within stratum using a biased-coin algorithm [64] embedded within the Target-D website which is housed on the secure National eResearch Collaboration Tools and Resources (Nectar) cloud which provides computing infrastructure to Australian researchers. Using restricted randomization within the stratum ensures that number of individuals is balanced between study arms within stratum and the stratification factors will be balanced in each study arm. The randomization will be triggered automatically within the Target-D website, after a participant has completed baseline measurements and the *diamond* CPT, thus ensuring allocation concealment.

#### Blinding

Due to the nature of the intervention, participants cannot be blinded to their treatment allocation. However, GPs will not be notified of their patients’ allocation to either intervention or comparison arm. No emergency unblinding of GPs is anticipated, including in the case of the research team alerting the GP to patient suicidality. As outcome assessment is conducted online, no blinding of outcome assessors is required. All study analyses will be conducted by a statistician blind to participants’ allocation; study arm allocation will be coded as A or B, with the code for the study arm revealed only after data are analyzed.

### Data collection

**Fig. 2:**
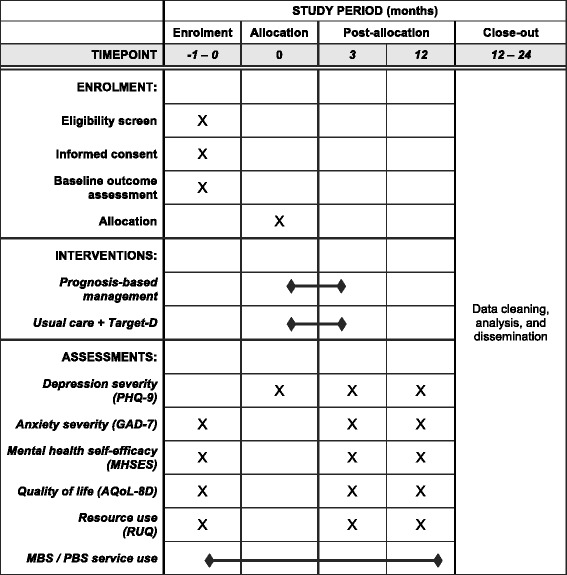
Schedule of enrolment, interventions and assessments. *PHQ-9 *Patient Health Questionnaire – 9; *GAD-7* Generalized Anxiety Disorder scale; *MHSES* Mental Health Self-Efficacy Scale; *AQoL-8D* Assessment of Quality of Life scale; *MBS* Medicare Benefits Schedule; *PBS* Pharmaceutical Benefits Scheme

Participant data will be collected from intervention and comparison arms using validated questionnaires on the Target-D website at screening, baseline and three and 12 months (Fig. 2). *diamond* CPT data will also be collected on the Target-D website. Participants will receive an automated email from the website at 80 and 358 days after *diamond* CPT completion with a unique link to the three-month and 12-month survey, respectively. Participants will be informed that if they decide to withdraw from the study, the data already provided will be retained and used in the analyses unless they request otherwise.

### Measures

Demographic characteristics, including age, gender, highest level of education, and employment status, will be assessed at baseline.

#### Primary outcome

Depressive symptom severity will be assessed at each timepoint using the PHQ-9. The PHQ-9 assesses the nine DSM symptoms of depression over the last two weeks using a 4-point Likert scale (where 0 = “not at all” and 3 = “nearly every day”). Total scores are in the range of 0–27, with suggested cut points of 5, 10, and 15 indicating mild, moderate, and severe depression, respectively [40]. The PHQ-9 is a validated diagnostic measure in primary care [65], with demonstrated efficacy and sensitivity as an outcome measure for treatment trials with a recommended Reliable Change Index [51].

#### Secondary outcomes

Self-efficacy will be measured using the Mental Health Self-Efficacy Scale (MHSES) [66]. The MHSES comprises six items that require respondents to rate on a 10-point Likert scale how confident they are in performing behaviors related to mental health self-care (from 1 = “not at all confident” to 10 = “totally confident”). Total scores are in the range of 6–60 and provide a unidimensional measure of self-efficacy; higher scores indicate greater levels of self-efficacy. The MHSES displays high internal consistency (Cronbach’s alpha = 0.91) and good construct validity, correlating well with measures of depression, anxiety, and functional impairment.

The seven-item Generalized Anxiety Disorder scale (GAD-7) will be used to assess anxiety [67]. The GAD-7 assesses the presence of anxiety symptoms over the past two weeks using a 4-point Likert scale. Scoring is similar to the PHQ-9; each item is scored from 0 to 3 (for a total possible score of 0–21), with cut points of 5, 10, and 15 corresponding to mild, moderate, and severe anxiety symptoms. The GAD-7 has excellent internal consistency (Cronbach’s alpha = 0.92) and test–retest reliability. Its construct, convergent, and discriminant validity are high; it correlates well with measures of depression and functioning (while assessing a distinct construct), as well as with other measures of anxiety.

Quality of life will be assessed at each time point using the Assessment of Quality of Life (AQoL-8D) [68]. This is a validated, reliable measure [69] that comprises eight dimensions (independent living, senses, pain, mental health, happiness, self-worth, coping, and relationships) that can be used to calculate quality-adjusted life years (QALYs) via a utility algorithm. The AQoL-8D has been shown to be sensitive to depressive symptom severity levels [69].

Cost-effectiveness of the intervention will be measured through assessment of health service use, effects on productivity, and calculation of QALYs. Health service use will be tracked using data extracted from the Australian Government Department of Health: the Medicare Benefits Schedule (MBS) that maintains information about visits to healthcare providers and diagnostic tests; and the Pharmaceutical Benefits Scheme (PBS) database of medications supplied on prescription. Participants will provide additional consent to access their MBS and PBS data. Other resource use not captured by these national databases, including the use of broader health and welfare services and effects on productivity (i.e. education and workforce participation), will be assessed via self-report using an adapted questionnaire developed by members of the research team and used in numerous other Australian mental-health intervention trials [70–72].

### Process data

To complement the outcome data collected as part of the RCT, a parallel process evaluation will be conducted in order to understand the context in which the outcomes were achieved. The evaluation will identify challenges of implementation and provide important guidance for future translation of trial findings, using the framework set out by the Medical Research Council [73]. The process evaluation will draw from data collected through a variety of sources, including but not limited to recruitment logbooks, interviews and surveys of GP and practice staff, intervention uptake and adherence data (as described above), and interviews with randomly selected participants (across both the two study arms and three depressive symptom severity groups). A comprehensive protocol for this evaluation will be published separately.

### Retention

To encourage retention at each study time point, non-responders will receive up to five reminders in total via phone, text, and email. These reminders will also provide the option of completing the baseline, three-month, or 12-month survey over the phone with an RA or being mailed a hard copy of the questionnaire to complete and return via reply paid envelope. At three and 12 months, participants who still do not complete the survey will be offered the option of completing the primary outcome measure (PHQ-9) alone. Outcome assessments may be completed in multiple sittings, with participants provided the option of saving their responses and returning later via a link emailed to them upon exiting the survey.

To acknowledge the time spent by participants and to further promote retention at three and 12 months, random draws for a $100 gift card will be conducted monthly for each follow-up survey, with all participants who completed the survey in the previous month eligible to receive a gift card. The selected participant will be contacted via phone and email. Participants will be advised of the draw in the initial email with their unique link to the relevant survey and in subsequent reminders.

### Data management

Participants will enter data directly into the Target-D website, which will store responses coded according to standard practice for each validated questionnaire. The website presents each item on a separate page to minimize the chance of items inadvertently being missed. Data integrity will be enforced through the use of forced or multiple choice items wherever possible; valid value and range checks will also be built into the website for free text fields where appropriate.

The coded study data will be downloaded weekly from the Target-D website, stored securely, and backed up regularly on a central password-protected University system. A data manager will check all data to identify and, where possible, resolve errors prior to analyses being conducted. Data will be kept for 15 years after study completion after which time they will be destroyed in accordance with University protocol [74].

The Research Electronic Data Capture (REDCap) secure software application [75] will be used to manage contact with participants and track progress through the study, with participant information transferred manually into REDCap from the Target-D study website. Both REDCap and the Target-D website are password-protected and housed on secure University servers; only the study team will have access to the identified data.

### Statistical methods

Descriptive statistics will be used to compare participant characteristics between the study arms, in total and stratified according to depressive symptom severity group. Linear mixed-effects model using restricted maximum likelihood with random intercepts for individuals will be used to estimate the difference in mean outcome between study arms at three and 12 months. All regression models will adjust for baseline outcome measure (where appropriate), stratification factors (practice, depressive symptom severity group) and time (baseline, three and 12 months), with a two-way interaction between study arm and time except baseline where means in the study arms will be constrained to be equal. Baseline variables strongly associated with the outcome that are found to be imbalanced between the study arms will also be considered for adjustment in the regression analyses. Estimated intervention effects will be reported as the difference in the means of the outcome between study arms (intervention-comparison), with 95% confidence intervals and *p* values. Similar regression analyses will also be used to compare the outcomes between intervention and comparison arms separately for each of the three depressive symptom severity groups. In a secondary analysis, we will investigate the intervention effect on individuals who would comply with their assigned treatment using a complier average casual effect (CACE) analysis [76]. A detailed analysis plan will be developed for the secondary and sensitivity analyses. All analyses will be performed using Stata 13.0 [77].

#### Missing data

Analyses will use an ITT approach, where participants will be analyzed in the study arm to which they were allocated [78]. In the first instance, we will implement strategies to minimize the missing outcome data, including the participant retention strategies outlined above. Reasons participants are lost to follow-up will be recorded. Sensitivity analysis will be used to assess the robustness of the missing data assumption.

#### Cost-effectiveness and cost-utility analysis

Incremental cost-effectiveness ratios (ICERs) will be determined (cost of intervention – costs of comparison/outcome of intervention – outcome of comparison) using the AQoL-8D to determine QALYs. ICERs using other important study outcomes (such as cost per remitted case) will also be determined. Variation will be determined by bootstrap and regression analyses and results presented in cost-effectiveness planes and acceptability curves. Sensitivity analyses will also be used to determine the impact of important study parameters (such as unit cost price variation). Dependent on trial results, modeling may also be used to extrapolate beyond the trial time horizon.

### Monitoring

The Target-D study will be monitored by the Steering Committee (SC) and a Data Monitoring Committee (DMC). The SC will comprise all named investigators and the project manager and will be led by the Chief Investigator. The SC will have biannual meetings to monitor recruitment progress, troubleshoot any areas of concern, ensure that the project is being conducted according to protocol, and identify additional training or support required by the research staff to facilitate the smooth running of the trial.

The DMC will comprise at least three members and be led by Professor Jon Emery, an experienced researcher independent of the research team. Collectively, DMC members will have clinical, research, and statistical expertise across primary care and mental health. Members of the DMC will be provided with a Charter outlining their scope of responsibilities (Additional file 5). The DMC will meet biannually to monitor trial processes and progress, and review complaints, harms, and adverse events. Adverse events may be serious or otherwise; the former are defined as those which “might be significant enough to lead to important changes in the way the [intervention] is developed” [79]. In light of the fact that the interventions used in the study are evidence-based, and all participants are linked in with health services, routine data collection will assess adverse events and no interim analyses or auditing are planned. All adverse events will be recorded (including relation to study, severity, potential for the event to have been anticipated, and action taken) and reported to the DMC. Serious adverse events will also be reported to the University ethics committee.

### Ethics and dissemination

The University of Melbourne Human Research Ethics Committee (HREC) has approved this study protocol (ID number 1543648). Collection of MBS and PBS data has been approved by the Australian Government Department of Human Services Information Services Branch (ID: MI3794). Approval from these two ethics committees applies to all study sites. Any substantive modifications to this protocol that affect the conduct or nature of the study will be submitted to the responsible HREC for approval prior to implementation.

Eligible patients will receive a plain language statement outlining the potential risks and benefits of participating in Target-D and give informed consent to participate in the study through the Target-D website. A copy of the plain language statement will also be provided via email. Consent will apply only to the current research study. Participants will be advised at the time of study consent that they will be asked for separate consent to collect their MBS/PBS data. Participants will subsequently receive a plain language statement regarding MBS and PBS data collection (Additional file 4) and a link to provide informed consent online. Participants will be advised that consenting to provide access to their MBS/PBS information is optional and will not affect their participation in Target-D. All information provided to participants regarding the collection of this data adheres to Australian Government requirements.

Confidentiality of participants will be protected by assignment of an identification number to each participant. Participants’ study information will not be released outside of the study without permission, except where maintaining confidentiality endangers the health or safety of the participant or someone else. Only investigators included in the original ethics applications or subsequent amendments will have access to the identified dataset.

### Declaration of interests

GA heads the Clinical Research Unit for Anxiety and Depression, which is home to This Way Up. As GA will not be involved in data analysis or interpretation, this interest will have no undue influence on the study findings. No other authors have competing interests to declare. During the trial, all authors will comply with their respective institution’s policies on conflicts of interest.

### Dissemination policy

Regardless of the magnitude or direction of effect, the results of this trial will be presented at relevant research conferences and as published articles in peer-reviewed journals. The study will be reported following the CONSORT and TIDier guidelines. Authorship eligibility guidelines at the respective institutions will be followed. The results of the trial will be communicated to participants via a trial newsletter and to the involved GP clinics via a personal visit and community reports. The findings from this trial have the potential to affect healthcare policy and will be reported to relevant government bodies. There are no plans to allow public access to the dataset or statistical code.

## Discussion

The burden of disease associated with depression is large and shows no sign of decreasing, despite significant investment in an array of effective treatments. One reason for this is suggested to be poor allocation of treatment, with both over-treatment for mild symptoms and under-treatment for severe symptoms common. Stepped care models, in which people receive the least time and resource intervention that will be effective, are posited as a solution to this mismatch; however, there is currently no systematic way of identifying which “step” of treatment an individual should be allocated to. In addition, mental health lags behind other fields of medicine in focusing on which step is appropriate given the person’s current symptoms, rather than their future course of illness.

We have therefore developed a new CPT which predicts depressive symptom severity at three months and provides an evidence-based treatment recommendation accordingly. In the Target-D trial, we will test whether using this tool to match individuals to treatment is a clinically effective and cost-efficient way of reducing depressive symptom severity, relative to usual care. If the Target-D model for depression management is efficacious and cost-effective, implementation into practice could reduce unnecessary treatment burden and improve allocation of treatment resources.

### Trial status

At the time of submission, patient recruitment to the Target-D trial is ongoing. The anticipated study completion date is July 2018.

## Additional files

Additional file 1: Trial registration data. Table presenting the World Health Organization Trial Registration Data Set. (PDF 89 kb)

Additional file 2: SPIRIT checklist. Table identifying where each SPIRIT checklist item is addressed in the manuscript. (PDF 59 kb)

Additional file 3: Target-D study sites. List of confirmed study locations at the time of submission. (PDF 28 kb)

Additional file 4: Informed consent materials. Plain language statements and consent forms. (PDF 1027 kb)

Additional file 5: Target-D Data Monitoring Committee Charter. Charter outlining the aims and terms of reference of the trial Data Monitoring Committee. (PDF 212 kb)

## Abbreviations

AQoL-8D: Assessment of Quality of Life (8 dimension version)
CACE: Complier average casual effect
CM: Case manager
CPT: Clinical prediction tool
DMC: Data monitoring committee
DSM: Diagnostic and Statistical Manual
GAD-7: Generalized Anxiety Disorder scale
GP: General practitioner
iCBT: Internet-based cognitive behavioral therapy
ICER: Incremental cost-effectiveness ratio
MBS: Medicare Benefits Schedule
MHSES: Mental Health Self-Efficacy Scale
Nectar: National eResearch Collaboration Tools and Resources
NPT: Normalization Process Theory
PBS: Pharmaceutical Benefits Scheme
PHQ-2: Patient Health Questionnaire (2-item version)
PHQ-9: Patient Health Questionnaire (9-item version)
QALY: Quality-adjusted life years
RA: Research assistant
RCT: Randomized controlled trial
REDCap: Research Electronic Data Capture
SC: Steering committee
UC+: Usual care plus Target-D
VicReN: Victorian Primary Care Practice-Based Research Network

## Footnotes

1 myCompass users can elect to receive helpful tips, facts, and motivational messages via SMS or email. The program also offers SMS/email reminders to facilitate symptom tracking and completion of homework activities; users can opt in and out of these services as they wish and can choose the frequency and timing of messages.
