# Supplementary material for: Target-D: a stratified individually randomized controlled trial of the diamond clinical prediction tool to triage and target treatment for depressive symptoms in general practice: study protocol for a randomized controlled trial
Source: Trials. 2017 Jul 20;18:342. doi: 10.1186/s13063-017-2089-y (PMC5520374; doi:10.1186/s13063-017-2089-y)
Supplement: Supplementary file 1 — Trial registration data. Table presenting the World Health Organization Trial Registration Data Set. (PDF 89 kb) [file 13063_2017_2089_MOESM1_ESM.pdf]

World Health Organization Trial Registration Data Set*

**Data category**	**Information**
Primary registry and trial identifying number	Australian New Zealand Clinical Trials Registry<br>ACTRN12616000537459
Date of registration in primary registry	27 April, 2016
Secondary identifying numbers	n/a
Source(s) of monetary or material support	National Health and Medical Research Council (NHMRC)
Primary sponsor	University of Melbourne<br>Grattan Street<br>Parkville<br>VIC 3010<br>Australia
Secondary sponsor(s)	n/a
Contact for public queries	AC via email
Contact for scientific queries	SF via email
Public title	The Target-D Study: An individually randomised controlled trial of a clinical prediction tool to triage and target treatment for depressive symptoms in general practice.
Scientific title	The Target-D Study: An individually randomised controlled trial of a clinical prediction tool to triage and target treatment for depressive symptoms in general practice.
Countries of recruitment	Australia
Health condition(s) or problem(s) studied	Depression
Intervention(s)	Active:<br>1. myCompass (self-help iCBT)<br>2. This Way Up (guided iCBT)<br>3. Collaborative care
	Comparison:<br>1. Usual care
Key inclusion and exclusion criteria	Inclusion: Aged 18-65; PHQ-2 score of 2 or more; regular access to computer with internet; able to comply with study intervention and assessments; if taking antidepressants, at least 1 month with the same medication
	Exclusion: Currently taking antipsychotic medication, currently seeing or planning to see a psychologist, currently accessing iCBT
Study type	*Type*<br>Interventional
	Design<br>Allocation: randomised; concealment via online algorithm<br>Masking: n/a<br>Assignment: parallel

	Purpose: treatment
Date of first enrolment	April 2016
Target sample size	570
Recruitment status	Recruiting
Primary outcome(s)	Change in depression symptom severity (PHQ-9)
Key secondary outcomes	Cost effectiveness, quality of life, anxiety, mental health self-efficacy

*For full Trial Registration Data see https://anzctr.org.au
