# Supplementary material for: Target-D: a stratified individually randomized controlled trial of the diamond clinical prediction tool to triage and target treatment for depressive symptoms in general practice: study protocol for a randomized controlled trial
Source: Trials. 2017 Jul 20;18:342. doi: 10.1186/s13063-017-2089-y (PMC5520374; doi:10.1186/s13063-017-2089-y)
Supplement: Supplementary file 3 — Target-D study sites. List of confirmed study locations at the time of submission. (PDF 28 kb) [file 13063_2017_2089_MOESM3_ESM.pdf]

Target-D study sites

The Target-D study will be conducted in at least 10 general practices in Victoria, Australia. At the time of submission, 8 sites had been confirmed. The location of these practices is as follows:

1. Essendon

2. Footscray

3. Preston

4. Ashburton

5. Mooroolbark

6. Noble Park

7. Altona Meadows

8. Surrey Hills
