# Supplementary material for: Target-D: a stratified individually randomized controlled trial of the diamond clinical prediction tool to triage and target treatment for depressive symptoms in general practice: study protocol for a randomized controlled trial
Source: Trials. 2017 Jul 20;18:342. doi: 10.1186/s13063-017-2089-y (PMC5520374; doi:10.1186/s13063-017-2089-y)
Supplement: Supplementary file 4 — Informed consent materials. Plain language statements and consent forms. (PDF 1027 kb) [file 13063_2017_2089_MOESM4_ESM.pdf]

Additional file 4. Informed consent materials

A. Study plain language statement

target D

Invitation

We'd like to invite you to take part in the Target-D Study.

Before you decide to take part, it is important for you to understand why the Target-D Study is being done and what is involved. Please take the time to read this information carefully and ask any questions if you wish.

If you are interested in taking part, please click on the link in your email and sign the consent form.

Thank you for taking the time to consider taking part in the Target-D Study.

Why Target-D?

Up to 55% of patients who see a GP have problems with stress, worries or depression. It is not always clear what treatment is right for individual needs. To help with this, researchers from the University of Melbourne have developed the Target-D toolkit which matches individuals to treatment that is right for them. The Target-D Study aims to evaluate the Target-D toolkit and its effectiveness as a tool for mental health care in GP clinics.

The Target-D Study has been funded by the National Health and Medical Research Council (NHMRC No: 1059863) and is led by Professor Jane Gunn from the Department of General Practice, The University of Melbourne.

What do I need to do?

- Answer about 15 questions in the Target-D toolkit. Depending on your answers to those questions, the toolkit will automatically allocate you into one of two groups. You do not get to choose which group. We call this "random allocation".

- Complete three surveys about your health and well-being, health service use and lifestyle at three time points over the next year.

Random allocation means that you will be assigned to a group by chance, like a flip of a coin.

The Target-D Team has no control over who will be allocated to which group.

Sharing information with your GP

The Target-D team may need to contact your GP to ensure that he or she is up to date about the treatment that you are receiving as part of the Target-D study.

If we need to share information with your GP will let you know about this beforehand.

We want to look at how Target-D affects health care use

You will be asked to fill out a consent form authorising the study access to your complete Medicare and Pharmaceutical Benefits Scheme (PBS) data at a later date

Medicare collects information on your medical visits and procedures, and the associated costs, while the PBS collects information on the prescription medications you have filled at pharmacies. The consent form is sent securely to the Department of Human Services who holds this information confidentially.

Whether or not you give us permission to access your Medicare information you can still participate in the study.

Your information will be de-identified to protect your privacy once we receive it from the Department of Human Services. We will remove all identifying information, such as your name, address and Medicare number and replace these details with a unique code.

Let's stay in touch

We greatly value your participation and to ensure that we can follow your progress throughout the study please let us know if you change address by emailing targetd-study@unimelb.edu.au.

If we lose contact with you we will use publically available listings to find your current postal and/or residential address (for example, Australia Post, Births Deaths and Marriages (Vic) and the Australian Electoral Commission).

What will be done to make sure my information is confidential?

Your privacy will be protected at all times and no individual participant will be identified or linked to the results.

Only the research team will have access to your information. We can only disclose information with your permission, or as required by law. All information will be stored on password-protected computers at the Department of General Practice, University of Melbourne for a minimum of 15 years before being destroyed.

In accordance with Australian and/or Victorian privacy and other relevant laws, you have the right to access and correct the information we collect and store about you. Please contact us if you would like to access your information.

HREC No.: 1543648.4 | V8 | Date: 25.09.2015

target↗D

What if I don’t want to participate or change my mind later?

Participating in this study is voluntary and you are free to withdraw at any time.

Your relationships with the University of Melbourne, your GP or GP clinic will not be affected. If you withdraw from the research project we will keep and use any information already collected unless you tell us not to.

What are the possible benefits of taking part in this study?

With your help, we can find out if the Target-D approach to emotional health and well-being is a better way of providing mental health care. Target-D will provide you the opportunity to reflect on your emotional health and well-being.

What are the possible risks?

We do not anticipate any risks, side-effects or discomforts.

Will I be informed of the results when the study is finished?

You will receive a summary of the research results at the end of the study. Results will reflect the whole group of participants, not your individual results. The results will not identify you in any way. The results of the project may be presented at conferences and published in professional journals.

Who can I talk to about this study?

If you have any concerns about the study or the way it is being conducted, and would like to speak to someone independent of the study, please contact:

Manager, Human Research Ethics, the University of Melbourne, Vic, 3010.

P: (03) 8344 2073;

F: (03) 9347 6739.

Target-D Ethics approval no: 1543648.

The Target-D Team

If you would like any more information about the study, please contact us on:

P: 0435 798 846

E: targetd-study@unimelb.edu.au

The Target-D Research Team and Study Investigators

Professor Jane Gunn, Associate Professor Catherine Mihalopolous, Professor Kelsey Hegarty, Dr Alishia Williams, Professor Leon Sterling, Dr Patty Chondros, Dr Sandra Davidson, Professor Gavin Andrews, Dr Victoria Palmer, Professor Elizabeth Murray, Professor Christopher Dowrick, Dr Gilles Ambresin, Professor Frances Griffiths, Ms Maria Potiriadis, Dr Caroline Wachtler, Ms Amy Coe and Dr Susan Fletcher.

B. Study consent (online)

THE UNIVERSITY OF MELBOURNE

target D

PARTICIPANT CONSENT FORM

- I voluntarily consent to take part in this research project.
- I understand that I can withdraw my participation at any stage of the project without explanation or prejudice.
- I have been informed that any relationship I currently have or will have in the future with the University of Melbourne, my GP, or my GP clinic will not be affected, whether I choose to participate in the study or not.
- I believe I understand the purpose, extent and possible effects of my involvement in this project.
- I have had an opportunity to ask questions and I am satisfied with the answers I have received.
- I understand that this project has been approved by the University of Melbourne Human Research Ethics Committee and will be carried out in line with the National Statement on Ethical Conduct in Human Research (2007, updated May 2015).
- I understand I will receive a copy of the Plain Language Statement, the Study Consent Form and Medicare Consent Form.
- I agree for a member of the Target-D research team to contact my GP about my depressive symptoms and the treatment I receive from Target-D.
- I understand that my postal and/or residential address may be obtained by the Target-D research team from publically available records.

STUDY CONSENT

Your full name

eg. John Smith

Today's date

10/10/2016

Your date of birth

SUBMIT

targetD

A randomised controlled trial of a clinical prediction tool

Your personal information is protected by law, including the Privacy Act 1988, and is collected by the Australian Government Department of Human Services. The collection of your personal information by the department is necessary for administering requests for statistical and other data.

Your information may be used by the department or given to other parties for the purposes of research, investigation or where you have agreed or it is required or authorised by law.

You can get more information about the way in which the Department of Human Services will manage your personal information, including our privacy policy at humanservices.gov.au/privacy or by requesting a copy from the department.

A sample of the information that may be included in your Medicare claims history:

Date of service	Item number	Item description	Provider charge	Schedule fee	Benefit paid	Patient out of pocket	Bill type
20/04/09	00023	Level B consultation	$38.30	$34.30	$34.30	$4.00	Cash
22/06/09	11700	ECG	$29.50	$29.50	$29.50		Bulk Bill

Scrambled ordering provider number*	Scrambled rendering provider number*	Date of referral	Rendering provider postcode	Ordering provider postcode	Hospital indicator	Item category
	999999A		2300		N	1
999999A	999999A	20/04/09	2300	2302	N	2

*Scrambled Provider number refers to a unique scrambled provider number identifying the doctor who provided/referred the service. Generally, each individual provider number will be scrambled and the identity of that provider will not be disclosed.

A sample of the information that may be included in your PBS claims history:

Date of supply	Date of prescribing	PBS item code	Item description	Patient category	Patient contribution (includes under copayment amounts**)	Net Benefit (this includes under copayment amounts**)	Scrambled prescriber number*	Pharmacy postcode
06/03/09	01/03/09	03133X	Oxazepam Tablet 30 mg	Concessional Ordinary	$5.30	$25.55	9999999	2560
04/07/09	28/05/09	03161J	Diazepam Tablet 2 mg	General Ordinary	$30.85		9999999	2530

Form category	ATC Code	ATC Name
Original	N05 B A 04	Oxazepam
Repeat	N05 B A 01	Diazepam

* Scrambled Prescriber number refers to a unique scrambled prescriber number identifying the doctor who prescribed the prescription. Generally, each individual prescriber number will be scrambled and the identity of that prescriber will not be disclosed.

** Under co-payments can now be provided for data after 1 June 2012

THE UNIVERSITY OF MELBOURNE

D. Medicare consent (online)

THE UNIVERSITY OF MELBOURNE

target↗D

MEDICARE CONSENT FORM

Important Information

- Complete this form to request the release of personal Medicare claims information and/or PBS claims information to the Target-D Study.
- Any changes to this form must be initialled by the signatory. Incomplete forms may result in the study not being provided with your information.
- By signing this form, I acknowledge that I have been fully informed and have been provided with information about this study. I have been given an opportunity to ask questions and understand the possibilities of disclosures of my personal information.

MEDICAL CONSENT

Salutation

Family name

First given name

Other given name

Second initial

Date of birth

Medicare number

Permanent address

Postal address (if different from above)

PARTICIPANT AUTHORISATION

I authorise the Department of Human Services to provide my:

- [ ] Medicare claims history
- [ ] PBS claims history

PARTICIPANT DECLARATION

Your full name

Today's date

SUBMIT
