# Supplementary material for: Target-D: a stratified individually randomized controlled trial of the diamond clinical prediction tool to triage and target treatment for depressive symptoms in general practice: study protocol for a randomized controlled trial
Source: Trials. 2017 Jul 20;18:342. doi: 10.1186/s13063-017-2089-y (PMC5520374; doi:10.1186/s13063-017-2089-y)
Supplement: Supplementary file 5 — Target-D Data Monitoring Committee Charter. Charter outlining the aims and terms of reference of the trial Data Monitoring Committee. (PDF 212 kb) [file 13063_2017_2089_MOESM5_ESM.pdf]

TARGET-D

DATA MONITORING COMMITTEE

CHARTER[1]

PROTOCOL: Target-D: A stratified individually randomized controlled trial of the diamond clinical prediction tool to triage and target treatment for depressive symptoms in general practice: Study protocol for a randomized controlled trial

SPONSOR OF PROTOCOL: The University of Melbourne

DATE: November 2016

[1] This Charter has been prepared using the DAMOCLES Study Group's 'A Proposed charter for clinical trial data monitoring committees: Helping them to do their job well' (Lancet 2005; 365; 711-22) and Chan et al's 'SPIRIT 2013 explanation and elaboration: guidance for protocols of clinical trials' (BMJ Research Methods and Reporting 2013; 346: e7586).

Introduction

This Charter is for the Data Monitoring Committee (DMC) for **Target-D.** Target-D is an individually randomised controlled trial (RCT) testing the *diamond* clinical prediction tool (CPT) – a novel tool developed by our team which predicts an individual's likely depression trajectory and provides a treatment recommendation matched to his or her likely depression severity at three months.

The Target-D RCT aims to test whether using this CPT is a cost-effective way of reducing depression symptoms, compared to usual care. The treatments recommended for each trajectory are described in the study protocol.

Target-D is registered with the Australia and New Zealand Clinical Trials Registry (CTRN12616000537459) and has ethics approval from The University of Melbourne Human Research Ethics Committee (1543648.1) and the Australian Government Department of Human Services (MI3794).

Funding body

Target-D (2014-2017) is funded by a project grant from the National Health and Medical Research Council (NHMRC; ID 1059863). The grant was awarded to and is coordinated by Professor Jane Gunn, Head of the Department of General Practice located in the Melbourne Medical School at the University of Melbourne.

Scope of this Charter

This Charter details the aim and terms of reference of the DMC for Target-D. It describes roles and responsibilities, membership and size, the frequency and format meetings, methods of providing information to and reporting from the DMC in the context of the Target-D trial.

Trial design

Target-D is a parallel comparison individually randomized controlled trial. Adult patients of participating general practices are invited to take part and complete all screening and recruitment procedures online. The *diamond* CPT is also completed online and simultaneously randomizes and triages patients. Randomisation is stratified by GP clinic and depression severity group; the allocation sequence is computer generated sequentially within stratum using a biased-coin algorithm. Figure 1 shows the trial design from the original Target-D protocol.

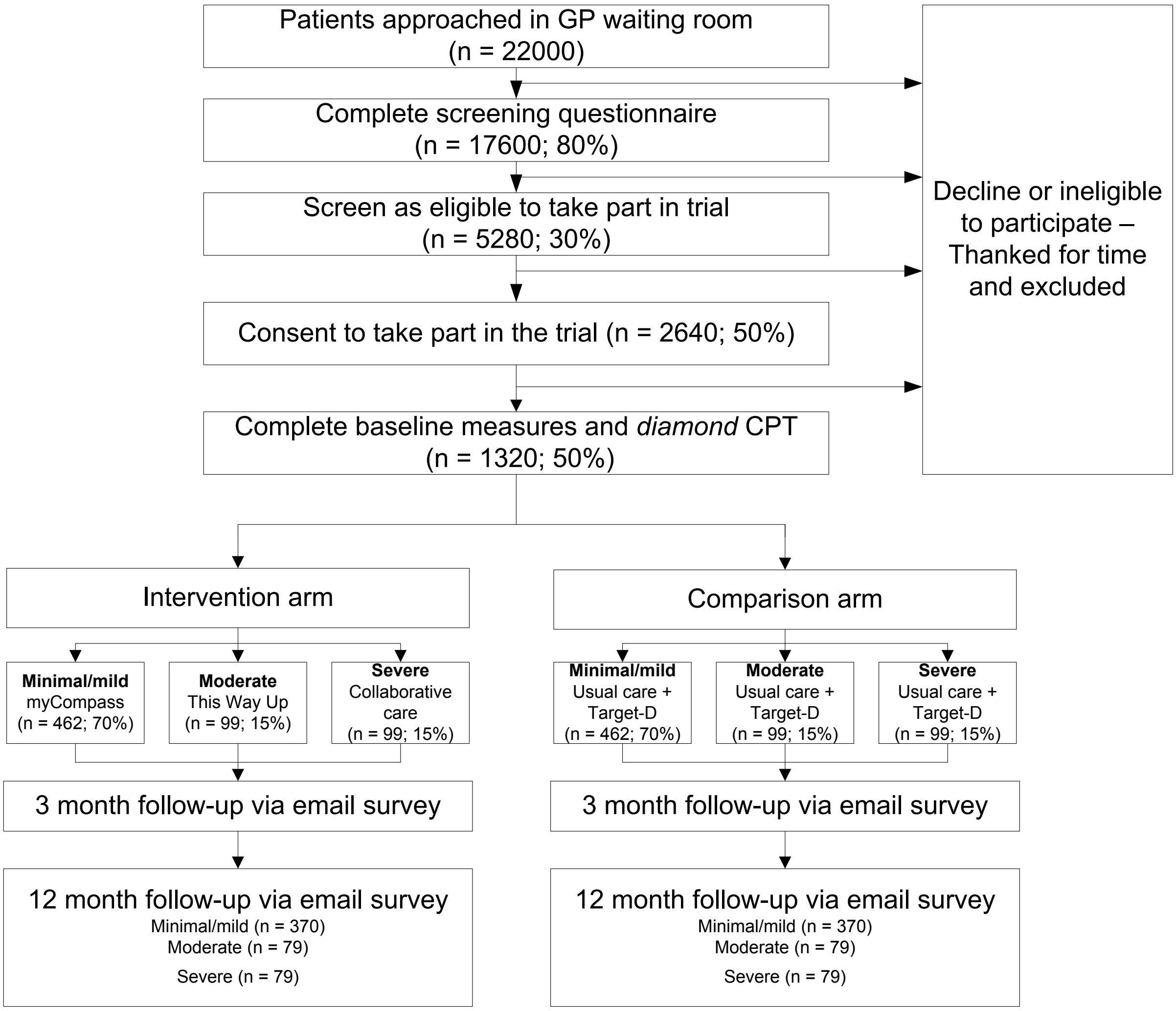

Figure 1. Target-D trial design

Roles and responsibilities

Aims of the committee

The DMC is responsible for safeguarding the interests of trial participants by assessing the safety of the interventions and monitoring the overall conduct of the trial. The DMC will provide advice to enhance trial integrity, recruitment and retention and procedures for data management and quality control. The DMC is advisory to the trial Steering Committee (SC) and project team.

Terms of reference

The DMC will be provided with biannual updates as to the progress of this trial and will, in turn, provide the SC with advice on trial conduct. In particular, the DMC will inform the SC if they determine that the results provide convincing evidence of the superiority or inferiority of one arm of the trial, whether for all participants or a subgroup, to the extent that the findings would substantively influence current practice or policy. The DMC will also inform the SC if they determine that continuation of the trial will not result in any clear outcome.

Roles of the DMC

The role of the DMC is to review trial progress and interim results. Specifically, the Committee will:

- monitor implementation of trial protocol;
- advise on protocol modifications suggested by investigators (e.g. to inclusion criteria, trial endpoints or sample size);
- advise on recruitment, retention and follow up issues;
- monitor sample size assumptions;
- advise on statistical analysis plan;
- consider adverse events and possible harms to study participants; and
- monitor compliance with previous DMC recommendations.

Before or early in the trial

DMC input into protocol

Potential DMC members will have the opportunity to review the trial protocol prior to joining the committee. The trial protocol will have undergone review by the funding body, clinical trials registry, and the University of Ethics committee and it is therefore a requirement of membership that individuals have no major reservations about the trial. Potential members that do are free to decline membership of the committee.

DMC meetings before the start of the trial

Given that DMC members are not required to have input into the trial protocol, there will not be a need for a meeting before the start of the trial. As per DAMOCLES[2], the first meeting will occur within one year of the first participant being recruited. Prior to the first meeting a 'dummy' report will be circulated to DMC members to provide an opportunity to become familiar with the reporting format.

Any issues specific to this study

No issues specific to the disease or treatment under study have been identified, nor any regulatory implications of DMC recommendations. The SC will advise the DMC of any such issues that arise during the course of the study.

DMC contracts

Members of the DMC will not have a contract formalizing their membership and clarifying issues around confidentiality and liability. Instead, DMC members will confirm in writing that they a) agree to be on the DMC and b) agree with the contents of this charter.

Composition

Membership and size of the DMC

There are 3 committee members for the DMC. Members represent a multidisciplinary mix of individuals with clinical and research expertise, across mental health, general practice, and statistics. All members are experienced in the conduct and monitoring of randomized clinical trials.

---

[2] Grant AM, Altman DG, Babiker AG, Campbell MK, Clemens F, Darbyshire JH, Elbourne DR, McLeer SK, Parmar MKB, Pocock SJ et al: A proposed charter for clinical trial data monitoring committees: Helping them to do their job well. Lancet 2005, 365(9460):711-722.

The members of the DMC for this trial are:

1. Professor Jon Emery (clinical trials and general practice)
2. Dr Nathan Alkemade (statistics and clinical psychology)
3. Dr David Pierce (general practice)

Conflicts of interest

Members of the DMC have been identified and selected because they do not have financial, scientific or regulatory conflicts of interest. DMC members will declare any competing interests they may have with the sponsor organisation or other relevant parties; members agree that if this changes they will notify the Chair. If significant conflicts of interest emerge during the course of the trial the member should resign from the DMC and the investigator team will reappoint a replacement.

DMC Chair

Professor Jon Emery has been selected by the SC as Chair of this DMC due to his expertise in general practice research and randomized clinical trials. He has extensive experience in serving on DMCs and in serving as committee chair. The role of the Chair is to summarise discussions and encourage consensus.

Responsibilities

DMC statistician

Dr Nathan Alkemade will provide independent statistical expertise and advice.

Trial statistician

Dr Patty Chondros will produce and/or oversee the analysis of data for reports to the DMC and will participate in DMC meetings where appropriate in order to guide the DMC through the reports and discuss statistical approaches to the final study analysis.

Project team

The trial manager (Dr Susie Fletcher) will produce the reports to the DMC and will participate in DMC meetings on request. The remainder of the project team will not usually be expected to attend but will be available to support the trial manager on non-confidential sections of DMC reports and to report to the DMC as necessary.

Steering committee

The Principal Investigator (PI: Prof Jane Gunn) will be available to attend DMC meetings when asked. The other members of the SC will not normally be expected to attend but may do so on request.

Relationships

The DMC functions in an advisory role only. The SC will consider recommendations made by the DMC but retains responsibility for all decisions.

DMC members are independent of the funding body and investigators. While both the SC and DMC include individuals associated with the University of Melbourne, they are geographically separate and no dependent relationships exist. The following diagram shows the relationship between the DMC and other committees and functional areas involved in the trial.

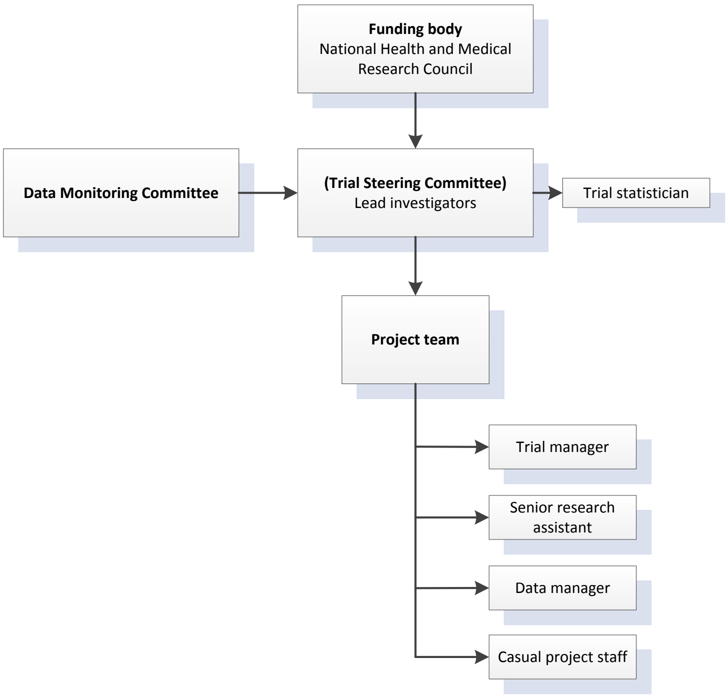

Figure 2. Target-D relationships

Payments to DMC members

DMC members will not be paid for their time on the committee. Members outside of metropolitan Melbourne will be reimbursed for travel and accommodation where necessary.

Organisation of DMC meetings

The Target-D DMC will meet bi-annually for up to two hours on each occasion. Meetings will be conducted in person at the Department of General at the University of Melbourne. Additional meetings may be held at the request of the DMC or SC in response to trial events.

In the first meeting of the DMC, members will be introduced to each other and the study protocol and discuss the Terms of Reference as stipulated within this Charter. The first meeting will also provide an opportunity for discussion and feedback on protocol implementation and recruitment and overall study progress. Members of the trial project team and SC may also attend this meeting.

Subsequent meetings will focus on updates about intervention implementation and maintenance, follow up, retention and attrition. After the conclusion of the participant recruitment and intervention phases of the trial, meetings will examine progress in the context of outcomes.

Each meeting may include a combination of open and closed agenda items; open items are those appropriate for trial staff and SC members to be present for, while closed items may involve presentation of data and results that should not be reviewed by investigators. Only DMC members and those they specifically request (e.g., the trial statistician) will be permitted to be present for discussion of closed items. For open items the DMC may be joined be members of the SC, project team, and/or other individuals as relevant.

Trial documentation and procedures to ensure confidentiality and proper communication

At least 2 weeks before each meeting, the trial manager will send DMC members a report with details on the trial progress, including recruitment, baseline characteristics of participants, available data, eligibility violations, adverse events or harms, withdrawals, and completeness of follow up. The trial manager and statistician are responsible for preparing these reports and will be overseen by PI Gunn.

All reports will include any reporting of adverse or harmful events that occurred since the previous ADMC meeting, including any relevant data analyses. Target-D adopts the definitions of adverse events and harms outlined by the CONSORT group, as detailed in Table 1 below.

Table 1. Definitions of adverse and harmful events in Target-D.[3]

**Adverse event**	Side effects that are harmful; these may be partially or entirely due to the intervention or totally unrelated.
**Serious adverse events**	Adverse events that, if suspected to be intervention-related, might be significant enough to lead to important changes in the way the intervention is developed (e.g., change in delivery, population, monitoring, consent).
**Harms**	The totality of possible adverse consequences of an intervention; they are direct opposite of benefits, against which they must be compared

The DMC members do not have the right to share confidential information with anyone outside the DMC, including the PI. The PI/project team will be responsible for circulating any external evidence from other trials/systematic reviews to the DMC members. The DMC will not be blinded to the intervention allocation.

All reports circulated to the DMC should be considered confidential and stored securely, whether in hard copy or electronically. After the trial is complete, DMC members should destroy all interim reports.

Within two weeks of each DMC meeting the Chair will provide written recommendations to the PI via email, copying in the trial statistician and trial manager. If no action is required, the DMC Chair's written report will indicate as such.

Decision-making

In making recommendations to the SC, the DMC may consider the following:

- No action required – the trial continues as planned.
- Stopping the trial (e.g., in case of clear benefit or harm, or new external evidence)
- Partially stopping the trial – e.g., stopping recruitment within a subgroup
- Extending the trial – either through extended recruitment, intervention period, or follow-up
- Changing the protocol in some other significant way.

[3] Adapted from Ioannidis JPA, Evans SJW, Gøtzsche PC, O'Neill RT, Altman DG, Schulz K, Moher D, for the CONSORT group. Better reporting of harms in randomized trials: An extension of the CONSORT statement. Annals of Internal Medicine 2004; 141: 781-788.

The DMC is asked to make decisions about the ethical, practical, statistical and financial implications of reports for the trial and make recommendations to the investigators. DMC members will provide advice on actions to be taken in the event of adverse or harmful events and review the procedures followed by the project team. All members of the DMC must be in attendance for decision-making, and members should make every effort to reach a unanimous decision. In cases where unanimity can not be reached, the Chair may request additional information from the trial management team, request advice from external parties, or recall the DMC at another time.

Specific issues relating to trial design

No specific issues relating to the trial design that might influence the DMC decision-making process. In the event that new issues arise during the trial, they will be brought to the attention of the committee and discussed as required.

Reporting

As noted above, the DMC will report its recommendations via email to the PI within 2 weeks of each meeting. DMC members will decide who will take minutes of each meeting; the minutes of each meeting will be reviewed and agreed on at the next meeting. Separate minutes may be required for open and closed agenda items if minutes are to be circulated to the project team or SC.

Serious disagreements between the DMC and SC will result in a joint meeting being held as soon as practicable, with the format and content of this meeting to be determined by the particular concerns to be discussed. An independent person will be asked to adjudicate this meeting and, in the event that the DMC and SC cannot reach agreement, will be responsible for making a decision about how to resolve the issue. The DMC and SC will be bound by this decision.

After the trial

After the conclusion of data collection the DMC will meet for the final time with the PI and project team to discuss any outstanding issues and advise on data interpretation.

The primary outcome paper for Target-D will include a list of DMC members' names and affiliations, unless they explicitly request otherwise. A brief summary of the timing and conclusions of DMC meetings will be included in the body of this paper. The DMC will be given the opportunity to read and comment on any publications prior to submission, any feedback provided will be acknowledged in print.

To maintain independence from the trial, DMC members external to the investigator group will not participate as authors in publications arising from trial data. If DMC members wish to disclose to the study team any issues that arose during deliberations, or any other concerns they have about their involvement in the trial, they may do so 12 months after publication of the primary results.
